# Social and environmental variables as predictors of mania: a review of longitudinal research findings

**DOI:** 10.1007/s44192-022-00010-5

**Published:** 2022-03-14

**Authors:** Sheri L. Johnson, Benjamin Z. S. Weinberg

**Affiliations:** Department of Psychology, University of California, 2121 Berkeley Way West, MC 2010, Berkeley, CA 94720-2010 USA

**Keywords:** Bipolar disorder, Mania, Social support, Family, Trauma, Life events

## Abstract

Considerable evidence suggests that psychosocial variables can shape the course of bipolar disorder. Here, though, we focus on the more specific idea that the social environment can predict the course of mania. We systematically review evidence from longitudinal studies concerning how social support, family interactions, traumatic life events, and recent life events relate to the age of onset, the frequency of episode recurrence, and the severity of manic symptoms. Although we find some evidence that the course of mania can be worsened by social environmental factors, the links are specific. Among social variables, some studies indicate that conflict and hostility are predictive, but more general social relationship qualities have not been found to predict mania. Some research indicates that childhood trauma, and recent life events involving goal attainment or sleep disruption can predict mania. Taken together, the profile of variables involving recent exposure that are most predictive include those that are activating, reward-related, or sleep-disrupting, which fits with general psychological hypotheses of behavioral activation and sleep disruption as important for mania. We discuss gaps in the literature, and we note future directions for research, including the need for more integrative, longitudinal research on a fuller range of social and biological risk variables.

## Introduction

Bipolar spectrum disorders are defined by manic symptoms of varying duration and intensity. The DSM differentiates three major types of bipolar disorders. Bipolar I disorder is defined by at least one lifetime episode of mania. Bipolar II disorder is defined by milder hypomanic episodes as well as episodes of major depression. Cyclothymia is defined by chronic fluctuations between depressive and manic symptoms that do not meet the criteria for hypomanic or manic episodes [1].

Bipolar disorder is well-established as a genetically driven illness. Nonetheless, social and environmental factors can influence the onset, course, and outcomes of this disorder. Here, we focus on how such variables may inform the course of manic symptoms, including onset, severity, and recurrence of manic or hypomanic episodes. Notwithstanding that depression is common among those with bipolar disorder [2] and is important for quality of life and functional outcomes within bipolar disorder [3, 4], we focus here on mania for a few reasons. First, manic symptoms are the defining feature of bipolar spectrum disorders. Second, many individuals with bipolar disorder never experience depression [2]. Third, given the evidence that depression and mania within bipolar disorder have separable genetic contributions [5], and fluctuate independently in an uncorrelated manner [6], there have been calls to consider whether mania and depression can be considered to be separable syndromes within bipolar disorder [7]. Taken together, it seems important to consider whether the social environment has direct contributions to the course of mania, and to examine which facets of the social environment are relevant.

We focus specifically on longitudinal studies that test whether socioenvironmental factors can predict change in mania parameters over time. This is not to minimize the bidirectionality of these effects—substantial evidence indicates that manic episodes exert profound influence on employment, finances, and relationships [3]. For example, bipolar spectrum disorders are related to high levels of functional impairment, unemployment, homelessness, and legal difficulties and to lower likelihood of becoming and staying married [8–11], and high rates of relationship dissatisfaction [12, 13]. Deficits in social support appear to worsen with manic episodes [14], with longer illness duration [15], with inter-episode subsyndromal symptoms [16, 17], and when individuals experience more internalized stigma concerning their bipolar disorder [18]. Given the evidence that difficult contexts can emerge consequent to symptoms, estimating the causal influence of social environmental factors on the course of disorder requires prospective research that controls for symptom levels at baseline.

In addition to the direction of effects, mania may shape how one interprets social circumstances, in that when manic, people may be less sensitive to negative information [19]. Where possible, we will highlight objective ratings of the social environment that have been used to supplement subjective ratings.

We consider several facets of mania here: age of onset, recurrence, and increases in the severity of manic symptoms. Where available, we consider evidence concerning the age of onset—a variable of particular merit in considering whether the social environmental features were present before manic episodes began. After onset, that the median time between manic episodes for those with bipolar I disorder is about one year [20]. Given the low frequency of mania recurrence, statistical power is often greater in analyses of symptom severity than for recurrence. Not surprisingly, then, many of the studies in the field focus on changes in symptom severity.

## Methodological approach

**Fig. 1:**
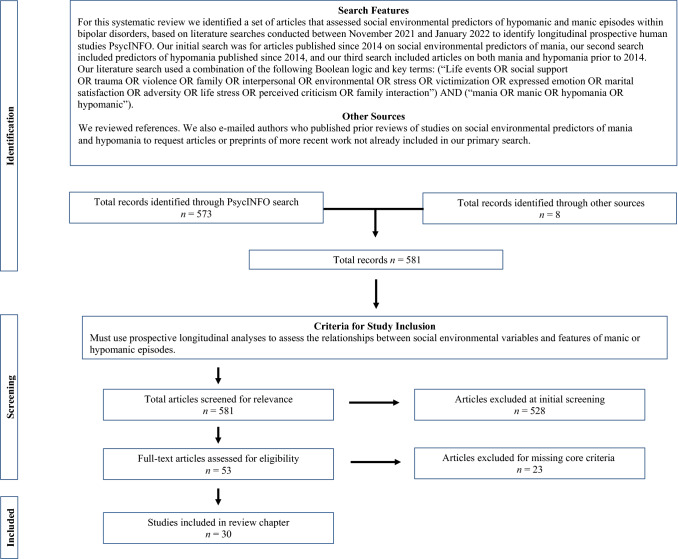
PRISMA-style flow diagram showing the search criteria, selection of studies, inclusion criteria, and exclusion rationale of studies for systematic review

PRISMA guidelines for systematic review were followed to conduct this review [21]. The PRISMA checklist is attached as Appendix A, and the PRISMA flowchart is included as Fig. 1. To consider the links of social environmental variables with the course of mania, we conducted a PsycINFO search for the terms (“Life events OR social support OR trauma OR violence OR family OR interpersonal OR environmental OR stress OR victimization OR expressed emotion OR marital satisfaction OR adversity OR life stress OR perceived criticism OR family interaction”) and (“mania OR manic”). In a second search, we sought articles that included the social terms, the words “hypomania OR hypomanic”, and were not covered in the first search. Both searches were restricted to longitudinal and prospective human studies. Because the first author had conducted a comprehensive review in 2016 [22], we first limited our searches to articles published since 2014. Upon editorial suggestion, we conducted a third search for articles published before 2014. The PsycINFO searches identified 573 articles (440 for mania, 37 additional articles for hypomania, and 96 for articles preceding 2014). B. W., S. J., and an assistant reviewed the articles to assess inclusion criteria, including whether it included (a) information specific to mania, including timing or presence of manic/hypomanic onset, presence or timing of manic/hypomanic relapse, and severity of manic/hypomanic symptoms over time; (b) social environmental variables (e.g., social, family, marital, trauma, adversity, or life events), and (c) analyses of how social environmental variables prospectively predicted mania-related variables. Environmental variables that were less clearly tied to the social domain, such as religiosity or exposure to in utero nicotine, were excluded from review, as were composite measures of functioning without specific indices such as family function or social support. S. J. and B. W. jointly reviewed articles where there were questions about inclusion. After review of these criteria, 53 articles were selected for systematic review. Most articles were excluded because they provided no longitudinal analyses of social variables. We also reviewed the references discussed in these articles, and we looked for relevant articles in previous reviews of life events [23, 24], expressed emotion [25] and broad predictors of mania [26]. We emailed authors who previously published regarding social factors in bipolar disorder to request articles, which yielded eight articles for potential inclusion. After more careful review, 30 articles (several of which contributed information about more than one social or mania parameter) were identified as assessing how social variables longitudinally predicted mania parameters; we describe findings from each of these below. Table 1 shows summary information for each of these studies.

**Table 1:** Summary of prospective studies of social environmental predictors of mania

Reference numbers	Author (year)	No. of bipolar spectrum participants	Bipolar diagnoses	Age at baseline Mean (SD)	Length of follow-up	Social Variables Assessed	Measures of social variables	Mania-related indices	Mania measure
[16]	Eidelman et al. (2012)	35	BP-I	34.66 (10.03)	1 month	Social support	ISEL	Change in symptom severity between baseline and 1-month follow-up	YMRS
[28]	Cohen et al. (2004)	52	BP-I	42.9 (12.5)	1 year	Perceived social support and life events	5-item social support scale and the UCLA ESI	(Hypo)manic relapse during follow-up	SCID
[29]	Johnson et al. (2000)	31	BP-I	42.29 (9.36)	9 months	Social support	ISEL	Change in symptom severity each month	BRMS
[31]	Weinstock & Miller (2010)	45	BP-I	39 (11.5)	1 year	Social support	ISEL	Change in symptom severity from baseline to 1-year follow-up	BRMS
[32]	Johnson et al. (2003)	94	BP-I (71) BP-II (23)	53.8	1 year	Social support	ISSI and ISEL	Hospitalization due to mania	YMRS
[33]	Koenders et al. (2015)	173	BP-I (121) BP-II (52)	49.9 (11.4)	2 years	Social support	SSL	Change in symptom severity across every 3 months	YMRS
[34]	Wicki & Angst (1991)	38	BP-II or NOS (21) Unipolar hypomanic (17)	Range: 28–30	9 years	Conflict and distress in relationships	SAS and SSIAM	Development of hypomanic episode by age 30	DSM-III
[36]	Sullivan et al. (2012)	58	BP-I (38) BP-II (6) BP NOS (14)	14.48 (1.60)	2 years	Family cohesion and family adaptability	FACES-II	Change in symptom severity every 3 months until the 12th month then every 6 months until the 24th month	Kiddie SADS
[38]	Townsend et al. (2007)	55	BP-I (53) BP-II (2)	Range: 5–17	8 weeks	Family function, communication, and problem-solving	FAD (parent rating)	Change in symptom severity between baseline and 8-week follow-up	YMRS
[39]	Kim & Miklowitz (2004)	125	BP-I (116) BP-II (9)	35.89 (10.36)	2 years	Expressed emotion	CFI	Change in symptom severity every 3 months until the 12th month then every 6 months until the 24th month	SADS
[40]	Yan et al. (2004)	47	BP-I	42.0 (13.1)	1 year	Expressed emotion	FMSS	Recurrence of manic or hypomanic symptoms preceded by 8 weeks without symptoms	SCID
[42]	Miklowitz et al. (2005)	360	BP-I (247) BP-II (93) BP NOS or cyclothymic or schizoaffective disorder (20)	40.6 (12.60)	1 year	Perceived criticism and distress from criticisms	PCS	Change in symptom severity between baseline and 1-year follow-up	YMRS
[43]	Geller et al. (2002)	89	BP-I	Range: 7–16	2 year	Maternal and paternal warmth and criticism per parent or child rating	PSS	Time to manic relapse	Washington University Kiddie SADS
[44]	Geller et al. (2004)	86	BP-I	Range: 7–16	4 year	Maternal and paternal warmth and criticism per parent or child rating	PSS	Time to manic relapse	Washington University Kiddie SADS
[45]	Geller et al. (2008)	115	BP-I	Range: 7–16	8 years	Maternal and paternal warmth and criticism per parent or child rating	PSS	Time to manic relapse	Washington University Kiddie SADS
[51]	Tijssen et al. (2010)	543 community participants without manic or hypomanic episodes	79 developed manic symptoms	Range: 14–17	8 years	endorsement of experiencing or witnessing a list of traumas	Self-rating	First onset of manic symptoms	M-CIDI
[52]	Daglas et al. (2014)	65	First episode psychotic mania	Range: 15–28	1 year	Direct personal physical, sexual, or emotional abuse or assault that would meet DSM-IV trauma criteria	Chart review	Mania severity at one-year follow-up, controlling for baseline	YMRS
[53]	Leverich et al. (2002)	373	BP-1 or BP-II	41 (12)	Mean of 2.8 years	Self-rated physical assault or abuse	Self-rating	Percent of time manic or hypomanic	YMRS
[54]	Pascual et al. (2020)	375	Bipolar spectrum disorder	17 Range: 8.7–25.8	Mean of 8.7 years	Parent or participant report of trauma	TES and brief interview with parent and child	Hypomanic episode	Adolescent LIFE
[55]	Gershon et al. (2013)	131	BP-I	40.9 (11.4)	2 years	Physical or sexual trauma	LEDS	Average mania severity across monthly follow-up interviews, controlling for baseline	BRMS interview
[56]	Meade et al. (2009)	90	BP-I and co-occurring substance use disorder	40.6 (11.7)	32 weeks	Two items covering lifetime physical or sexual abuse	Self-rating	Number of weeks manic or hypomanic	LIFE
[57]	Nolen et al. (2004)	258	BP-I (196) BP-II (53) BP NOS or schizoaffective disorder (9)	43.6 (10.8)	1 year	Serious physical, verbal, or sexual abuse as a child or adult	Self-rating	Average daily severity of mania	NIMH Life Chart
[61]	Johnson et al. (2008)	125	BP-I	Min: 18	2 years	Goal-attainment life events	LEDS	Change in symptom severity across each month	BRMS
[62]	Hosang et al. (2012)	96	BP = I or BP-II Mean number of depressive episodes: 13.87 (22.00). Mean number of manic episodes: 11.81 (20.94)	51.23 (9.73)	8 months	Life events	LEDS interview	Change in symptom severity between baseline and 4-month follow-up	SRMI
[63]	Johnson et al. (2000)	43	BP-I	Range: 18–65	2 years	Goal-attainment life events	LEDS	Change in symptom severity between baseline and 2 months after goal-attainment life event	BRMS
[64]	Nusslock et al. (2007)	68	BP-II (9) Cyclothymic (14) Both BP-II and cyclothymic (45)	22.1 (1.8)	6 weeks	Goal-attainment life events	LEI	Change in symptom severity between baseline and exam period 6 weeks after	SCID
[65]	Francis-Raniere et al. (2006)	106	BP-II (67) Cyclothymic or BP NOS (39)	19.91 (0.21)	4 months	Life events	LEI and LES	Time to onset of hypomanic peak	SADS
[66]	Gerstein (2011)	112	BP-I, BP-II, or cyclothymic	19.66 (1.83)	3 years	Life events	LES and LEI	Change in symptom severity every 4 months	SADS
[67]	Staufenbiel et al. (2014)	71	BP-I (54) BP II or BP NOS (17)	Med: 52 IQR: 43–62	3 months	Social support and life events	SSL and IRLE	Number and severity of symptoms across visits	YMRS
[77]	Sylvia et al. (2009)	101	BP-II (64) Cyclothymic (37)	19.8 (1.8)	1 year	Sleep loss	LES	Change in symptom severity at every 4-month follow-up	SADS

BIS/BAS, Behavioral Inhibition System/Behavioral Activation System Scale; BP, bipolar disorder; BRMS, Bech-Rafaelsen Mania Rating Scale; CFI, Camberwell Family Interview; DSM, Diagnostic and Statistical Manual of Mental Disorders; FACES-II, The Family Adaptability and Cohesion Evaluation Scale-II; FAD, Family Assessment Device; FMSS, The Five-Minute Speech Sample; IRLE, Interview for Recent Life Events; ISEL, Support Evaluation List; ISSI, Interview Schedule for Social Interaction; LEDS, Bedford College Life Events and Difficulties Schedule; LEI, Life Events Interview; LES, Life Events Scale; LIFE, Longitudinal Interval Follow-Up Evaluation; M-CIDI, Munich-Composite International Diagnostic Interview; PCS, Perceived Criticism Scale; PSS, Psychosocial Schedule for School-Age Children—Revised; SADS, Schedule for Affective Disorders and Schizophrenia; SAS, Social Adjustment Scale; SCID, Structured Clinical Interview for DSM Disorders; SRMI, Self-Report Mania Inventory; SSIAM, Structured and Scaled Interview to Assess Maladjustment; SSL, The Social Support List; TES, Trauma Events Screen; UCLA ESI, Episodic Stress Interview; YMRS, Young Mania Rating Scale

## Evidence concerning the social environment as a predictor of the course of mania

We begin by discussing the influence of social support, then discuss the more specific domain of family functioning. After discussing this literature on relationships, we consider the effects of traumatic events and life events.

### Social support

The term “social support” has been used to refer to emotional and material resources provided by a person’s social network. In general, social support has been found to be more predictive than social network size for mental health outcomes [27].

Relative to controls, people with bipolar disorder report low availability and adequacy of social support, even between episodes of illness [28]. In prospective studies, social support has been found to predict increases in depression over time [29–31]. In contrast, multiple indices of social support—including indices of social strain, satisfaction with social support or the degree of behavioral support received –have been shown to be unrelated to manic symptom severity, to manic relapse, or to hospitalization for mania [16, 29, 31–33]. In contrast, in the Zurich study, among 38 participants who developed hypomanic episodes by age 30, endorsement of conflict with friends at age 22, preceding onset, was significantly higher than among the 377 individuals without hypomanic episodes [34]. Thus, based on a single study, conflict within relationships appears to be predictive of hypomanic episodes, but findings of multiple studies indicate that general social support variables are not predictive of the course of manic symptoms.

### Family factors

Multiple facets of family relationships have been considered in relation to bipolar disorder. These include questionnaires and observer ratings to assess family function or specific facets of family function. One domain that has received particular attention is expressed emotion (EE), which is defined by observer ratings of criticism, hostility or emotional over-involvement from family members towards the person with bipolar disorder during a standardized interview in which the patient is not present [35].

Multiple facets of family function have not been found to predict the course of mania, despite significant effects on depression course observed in most of these studies. More specifically, observer ratings of family functioning were not found to predict changes in mania over a 1-year period among persons with bipolar I disorder [31]. Similarly, parent and child ratings of family cohesion and family adaptability were not found to predict significant change in manic symptoms among adolescents with bipolar disorder followed for 2 years [36]. Among youths ages 5 to 17 diagnosed with bipolar spectrum disorder, self-ratings of family function, communication, and problem-solving on the Family Assessment Device [37] did not predict change in interview-based mania ratings [38]. EE has also been found not to significantly predict manic symptoms over time [39, 40].

A different pattern emerges when researchers have examined more specific aspects of family function, in that mirroring the social support literature, family conflict and parental criticism do appear to be predictive of mania in several studies. In a study of adolescents diagnosed with bipolar disorder enrolled in a treatment trial and followed for two years, parent and adolescent reports of family conflict both predicted more prolonged manic symptoms, and improvement in family conflict was associated with greater decline in manic symptoms [36]. Similarly, parental criticism, as rated by observers watching a standardized family interview, was the only facet of EE found to predict greater manic symptoms among children diagnosed with bipolar disorder [41]. In one study, self-ratings of *severity* of *distress* over, but not *severity *of, parental criticism predicted change in manic symptoms [42].

Effects of family conflict were not consistent when considered in one younger sample of preadolescent and early adolescent youth who had experienced manic episodes. In that sample, when either mothers or children reported a low degree of maternal warmth toward the child, this predicted greater risk of relapse of manic/hypomanic symptoms within a 2-year [43], 4-year [44], and 8-year follow-up [45]; in the same study, neither paternal warmth nor maternal criticism predicted relapse. As noted above, statistical power to examine relapse timing may be lower than the power to examine severity of manic symptoms, which could help explain the null findings for maternal criticism. Alternatively, the studies above, which focused on adolescents, might have captured a more ideal developmental period for studying family conflict, given the naturalistic increases in parent–child conflict during that time period. Given positive effects for warmth in this study but not in other research, though, further research is warranted.

Taken together, findings of several studies indicate that parent–child conflict, parental criticism, and distress over parental criticism may contribute to more severe (hypo)manic symptoms among adolescents with bipolar spectrum disorders. The findings regarding conflict and criticism are distinct from a set of null findings regarding more general facets of relationships (with the exception of one study identifying maternal, but not paternal warmth as a predictor). One intriguing possible explanation for this specific profile of effects is that conflict and criticism are agitating, which could relate more directly to triggering high activation symptoms of mania.

### Childhood Traumatic events

About half of people diagnosed with bipolar disorder report a history of severe childhood abuse [46], and childhood trauma exposure is correlated with many different indicators of severity of bipolar disorder [47]. More specific to mania, large-scale studies indicate that among those diagnosed with bipolar spectrum disorder, early abuse and trauma are correlated with an earlier age of onset [46, 48], and more severe manic symptoms during adolescence [49].

Trauma has been suggested to influence inflammatory responses, which in turn, are implicated in symptom levels. Consistent with this idea, in one study, a genetic polymorphism tied to innate immune response, the TT genotype of the toll-like receptor 2 (TLR2) rs3804099, implicated in innate immune response to pathogens, was related to a stronger effect of self-rated sexual abuse on age of onset in bipolar disorder [50].

Some prospective research on trauma in bipolar disorder is available. In the general population, trauma did not significantly predict manic symptoms over time, highlighting the import of studying samples that are vulnerable to mania [51]. When researchers have focused on those who have already experienced manic episodes, trauma was found to predict more rapid mania recurrence among those with first episode psychotic mania [52] and greater likelihood of hypomanic symptoms among adolescents diagnosed with bipolar spectrum disorder [53]. Findings from the Course and Outcome of Bipolar Youth (COBY) study are particularly notable, as researchers completed interviews to assess symptoms and trauma within a large sample of 375 youths and young adults diagnosed with bipolar spectrum disorder, and they assessed trauma with parent and self-report measures [54]. Controlling for covariates, lifetime history of abuse per parent and child report was related to 1. 4 times the risk of hypomanic episodes over time but was not related significantly to full-blown manic episodes.

The effects of trauma may be particularly pernicious when exposure occurs during childhood as compared to adulthood. When researchers coded trauma exposure across the lifetime including recent trauma, trauma exposure did not predict significant changes in manic symptoms [55]. In a second study, lifetime trauma exposure also did not predict manic episodes, although did predict the likelihood of depressive symptoms during manic episodes (mixed episodes) [56]. In one study which directly compared childhood vs. adult abuse, childhood, but not adulthood, physical abuse prospectively predicted severity of mania across a one-year follow-up [57]. Taken together, three studies indicate that early trauma exposure appears meaningful for the prediction of hypomanic or manic episodes among those with bipolar spectrum disorder, in samples defined by bipolar spectrum disorders and by psychotic mania, with findings of one study suggesting effects specific to hypomania (as compared to mania).

### Recent life events

Although many studies have focused on life events in bipolar disorder, most rely on self-report scales, which have lower validity and reliability than interview measures do [58]. It also is difficult to rule out the possibility that prodromal symptoms contributed to stressors when using self-report measures. For example, a person who develops heightened confidence and impulsivity during a manic episode may spend large sums of money, creating significant financial stress. Thus, the causality of findings that stress levels and relationship problems are correlated with manic severity [59] can be difficult to interpret. Here, then, we focus on studies that use the Bedford College Life Event and Difficulty Schedule [60] or other interview measures to differentiate life events triggered by illness characteristics from those that appear to be independent of illness.

Prospective studies using life event interviews indicate that independent, negative life events, and chronic recent stressors predict increases in bipolar depression and delayed recovery from episodes of depression, but not mania [28, 55, 61]. Within negative life events, loss and danger events both related to depressive, but not manic symptoms, across an 8-month follow-up period [62].

Although negative life stressors do not appear related to mania, other facets of life events do appear related. For example, findings of several studies indicate that life events which involve goal attainment, such as getting married, having a child, or completing a degree, precede increases in manic symptoms [63]. In an initial study, goal-attainment life events predicted increases in manic but not depressive symptoms after controlling for baseline symptoms among persons with bipolar I disorder. This effect is observed despite removing events that are potentially caused by symptoms. These findings have been replicated in bipolar I disorder [61] and bipolar spectrum disorder [64]. Goal attainment events, which involve motivated pursuit of a desired goal, appear more powerful than more general positive events [63]. Among those with bipolar spectrum disorder who experienced life events related to goal pursuit, those with more perfectionistic, goal-striving cognitive styles showed more increase in hypomanic symptoms and more frequent hypomanic episodes during a three-year follow-up period [65, 66]. In each of these studies, the effects of goal attainment events appeared to be specific, in that such events were not significantly related to change in depressive symptoms.

Some research has examined mechanisms through which life events involving goal attainment become translated into symptoms. Unlike negative life events, goal attainment life events do not appear related to hair cortisol levels [67]. In a daily monitoring study, people with bipolar disorder were found to become more active and energized, relative to controls, after initial progress toward a goal [68]. Increases in goal engagement (setting new goals and spending time pursuing goals) have been found to predict increases in manic symptoms over several months among those diagnosed with bipolar I disorder [69]. Hence, increases in activity and goal pursuit may at least partially mediate the effects of goal attainment on mania. Some work has shown that national profiles of cultural values related to goal striving and individualism correlate with the prevalence levels of bipolar diagnoses [70]. This work fits with a broader set of findings on increased activity of the behavioral activation system as implicated in the onset of manic episodes [71].

Mania also has been found to relate to life events that involve schedule disruption. The influential social *zeitgeber* theory proposes that people with mood disorders may be particularly vulnerable to events that disrupt social rhythms, as these have the potential to trigger disturbances in sleep [72]. This builds from the extensive evidence that those with bipolar disorder show dysregulation in their sleep and circadian rhythm profiles [73]. Social rhythms are defined as daily routines that involve consistent timing (e. g., routines in timing of sleeping, eating, exercise, and social interactions) and thus help entrain natural circadian rhythms. Those with bipolar disorder have been found to have diminished social rhythms in their daily routine, and lower levels of social rhythm have been shown to predict the onset of bipolar spectrum disorder [74]. According to social zeitgeber theory, events that disrupt social rhythms—such as travel across time zones, loss of a spouse, or unemployment—can trigger disturbances in sleep and the sleep–wake cycle, which in turn, precipitate relapse among people with bipolar disorder [72]. Consistent with theory, social rhythm disrupting events have been shown to be more common before manic than depressive episodes within bipolar disorder [75, 76]. In one prospective study, events that specifically disrupted sleep predicted increases in hypomanic symptoms, whereas more general schedule disruptions due to life events were not predictive of change in hypomanic symptoms [77].

## Conclusions

A growing number of studies have examined the effects of the social environment on the course of mania within bipolar disorder. Findings indicate that more general facets of relationship quality, such as measures of social support, family function, or expressed emotion, do not appear to predict change in manic symptom severity over time in most studies, and yet findings of several studies indicate that conflicts with friends and family and parental criticism predict change in (hypo)manic symptoms. Measures of recent negative life events do not predict increases in mania over time, and yet findings of several studies indicate that early trauma exposure predicts increases in manic symptoms and greater risk of hypomanic recurrence over time. Goal attainment life events have been found to predict increases in manic symptoms in several studies, and cultures with higher rates of goal striving and individualism show higher prevalence of bipolar disorder. Consistent with the large literature linking sleep and circadian disruption to mania, sleep-disrupting life events have been found to predict change in manic symptoms within one study. Overall, then, findings of this small literature indicate that specific aspects of the social environment appear to be important for predicting the course of mania, including family conflict and criticism, early trauma, and recent life events involving goal attainment or sleep disruption.

The profile of variables that appear predictive, as compared to those with null effects, suggests some intriguing future directions. The relatively larger effects for early trauma may reflect the import of events occurring during critical periods for brain development [78]. In considering the influence of more recent social experiences, specificity appears to matter. Speculatively, social relationship variables that could trigger agitation may be important to consider. Within the domain of life events, mania effects appear specific to life events related to activation and sleep disruption. Taken together, the profile of these effects suggests the potential importance of early exposure and of recent risk variables that are that are activating, reward-related, or sleep-disrupting. This is intriguing, given that two major hypotheses of psychological contributors to mania involve dysregulation in the behavioral activation system involved in reward pursuit—believed to involve hyperactivation as a consequence of excessive goal pursuit [71] and dysregulation in sleep and circadian rhythms [73]. Nonetheless, it is important to note that some of the social variables predictive of mania, such as trauma and sleep disruption, also appear transdiagnostically important; without further research, it is difficult to know whether any mania-specific mechanisms are involved.

## Limitations and future directions

Despite evidence for the import of the social environment for mania onset, recurrence, and severity, only a small number of studies are available to consider each social domain covered here, and we were unable to identify any data on some key facets of the social environment. For example, although cross-sectional evidence suggests that being married is correlated with diminished symptoms, including fewer manic episodes among men [79]**,** we were unable to identify longitudinal studies of marital status or marital relationship quality in relation to mania. Major national differences in prevalence also suggest the likelihood of strong cultural influences on the course of bipolar disorder [80]. More work is warranted to understand the mechanisms driving these cultural effects.

More work is also needed to consider whether findings generalize across forms of disorder, and the range of mania-related symptom outcomes that can be predicted. Many of the samples studied include individuals with a broad range of bipolar spectrum disorders, and analyses did not differentiate those with bipolar I from those with bipolar II or cyclothymic disorders. Given increasing evidence for genetic differentiation in the forms of disorder [81], examining effects within subtypes will be an important goal for future research. It is also the case that most studies were only statistically powered to examine change in the level of manic symptoms, and not the onset or recurrence of disorder. Hence it will be important for future studies to consider more specific analyses of the types of disorder and the types of manic outcome variables that are tied to these social variables.

We also express caution, in that interpersonal domains are intricately inter-related, and most studies reviewed here examine only a small slice of the interpersonal world. As one example, within couples where one partner was diagnosed with bipolar spectrum disorder, higher relationship satisfaction predicted higher perceived social support, and vice versa [82]. Parental criticism of adolescents with bipolar disorder was also tied to greater family conflict [41]. Broader batteries encompassing multiple facets of the social environment will be important for disentangling these effects.

Beyond the need to disentangle social variables, there is a profound need to integrate social and biological risk factors in studies of bipolar disorder, given the estimates of 80% or higher heritability for bipolar disorder [5], and the evidence that exposure to early trauma can trigger long-lasting neural changes [78]. Findings of one study show that trauma effects on mania may be heightened among those with genetic polymorphisms related to inflammation [50]; these integrative studies are particularly important.

We are optimistic that currently underway studies will fill many of the gaps in current knowledge of social variables. That is, promising work has begun with large samples of those at risk for bipolar disorder, to gather self-rated measures of trauma, life stress, social support and marital satisfaction along with other key biological and personality risk variables to prospectively track conversion into the disorder [83, 84]. We urge careful attention to specific dimensions in analyses of those datasets.

## Implications

The early evidence described here that the social environment can shape the severity of mania fits with the evidence that psychological interventions, when added as adjuncts to medication, can help reduce symptoms of bipolar disorder. Large-scale research indicates that cognitive behavioral, psychoeducation, and family interventions can improve outcomes in bipolar disorder, including mania [85]. In keeping with the treatment outcome findings, several national standards for the treatment of bipolar disorder suggest providing psychotherapy as standard adjunctive care for bipolar disorder [86, 87].

With greater attention to the nature of diagnoses and manic outcomes, current findings could also inform treatment development. Given that mania severity is tied to more specific facets of the social environment, such as conflict or criticism rather than global relationship quality, or sleep disruption rather than general life events, one goal might be to examine how well psychosocial interventions can address these narrower facets of the social environment. To date, some work suggests that family interventions may be particularly helpful in reducing the duration of depressive and manic episodes among those with more serious family impairment [88] and those experiencing higher EE [89]. Research, though, suggests that family-focused therapies more readily enhance positive communications than reduce EE among families of those with bipolar disorder [90, 91]. Might a treatment focused specifically on family criticism provide more robust mania effects? Given that distress in response to family criticism can predict change in manic symptoms, work showing that psychological intervention can reduce amygdala reactivity to emotion stimuli provides one promising approach to this issue [92]. We also reported findings from a single study showing a role of events disrupting sleep. Although in need of replication, this finding has intriguing parallels with research documenting that intervention targeting insomnia can reduce manic symptoms [93]. Targeted intervention work provides the unique opportunity to examine experimentally how change in these key risk factors can promote better outcomes.

In sum, the small research base on social environmental predictors of mania suggests a profile of findings with intriguing clinical implications. Despite the need for more replication, for more careful examination of generalizability across bipolar diagnoses, and for integrative, prospective research, there is evidence that the social environment has an influence on the onset, severity, and recurrence of mania. That is, multiple studies indicate that early adversity and recent social challenges of patients could help shape the severity of mania. Several interventions are available to address social risk factors, including interpersonal/social rhythm psychotherapy and family-focused therapy. By considering social risk variables, clinicians can consider how to tailor treatment to address these key issues.

## Data Availability

Not applicable.

## Appendix A: Prisma checklist

**Table:**

Section/topic	#	Checklist item	Location(s) reported
*Information sources and methods*			
Database name	1	Name each individual database searched, stating the platform for each	PsycINFO
Multi-database searching	2	If databases were searched simultaneously on a single platform, state the name of the platform, listing all of the databases searched	N/A
Study registries	3	List any study registries searched	N/A
Online resources and browsing	4	Describe any online or print source purposefully searched or browsed (e.g., tables of contents, print conference proceedings, web sites), and how this was done	N/A
Citation searching	5	Indicate whether cited references or citing references were examined, and describe any methods used for locating cited/citing references (e.g., browsing reference lists, using a citation index, setting up email alerts for references citing included studies)	Cited references were examined for relevant work to be included in the review. Email alerts were also enabled for new studies referencing cited work
Contacts	6	Indicate whether additional studies or data were sought by contacting authors, experts, manufacturers, or others	Authors of previous work were contacted to request relevant articles
Other methods	7	Describe any additional information sources or search methods used	Previous reviews of social factors related to mania were examined for key empirical work to be included
*Search strategies*			
Full search strategies	8	Include the search strategies for each database and information source, copied and pasted exactly as run	The following queries were performed within PsycINFO Primary search query: FIELD 1: Life events OR social support OR trauma OR violence OR family OR interpersonal OR environmental OR stress OR victimization OR expressed emotion OR marital satisfaction OR adversity OR life stress OR perceived criticism OR family interaction FIELD 2: Mania OR Manic We subsequently conducted a search using FIELD 1 and setting the second search query to “hypomania” OR “hypomanic”
Limits and restrictions	9	Specify that no limits were used, or describe any limits or restrictions applied to a search (e.g., date or time period, language, study design) and provide justification for their use	Search was restricted to longitudinal prospective human studies as this was the focus of our review
Search filters	10	Indicate whether published search filters were used (as originally designed or modified), and if so, cite the filter(s) used	N/A
Prior work	11	Indicate when search strategies from other literature reviews were adapted or reused for a substantive part or all of the search, citing the previous review(s)	Five review chapters were examined for integration with the present review [22–26]
Updates	12	Report the methods used to update the search(es) (e.g., rerunning searches, email alerts)	Searches were rerun biweekly and email updates were configured for new articles matching the specified search criteria
Dates of searches	13	For each search strategy, provide the date when the last search occurred	The last primary search occurred 12/28/21. Authors of previous work were contacted 11/11/2021
*Peer review*			
Peer review	14	Describe any search peer review process	B. W. and an assistant reviewed all search results and then S. J. reviewed results. Any uncertainties were discussed between B. W. and S. J
*Managing records*			
Total Records	15	Document the total number of records identified from each database and other information sources	8 articles were received from authors who previously published work on social factors in bipolar disorder. Our initial PsycINFO search for articles published in or after 2014 produced 440 records for mania and 37 for hypomania. 96 articles were identified preceding 2014 for both mania and hypomania. After manually removing records that did not use longitudinal methods or have direct relevance to social environmental predictors of mania, 53 articles were identified for more systematic review. After the more careful review, 30 articles were identified as longitudinally using social variables to predict mania parameters
Deduplication	16	Describe the processes and any software used to deduplicate records from multiple database searches and other information sources	As PsycINFO was the only database used, no deduplication was necessary
